# The new European Medicines Agency guideline on antidepressants: a guide for researchers and drug developers

**DOI:** 10.1192/j.eurpsy.2023.2479

**Published:** 2023-12-15

**Authors:** Florence Butlen-Ducuing, Marion Haberkamp, Georgios Aislaitner, Ewa Bałkowiec-Iskra, Taina Mattila, Marika Doucet, Marta Kollb-Sielecka, Pavel Balabanov, Ann-Kristin Leuchs, André Elferink

**Affiliations:** 1 Office of Therapies for Neurological and Psychiatric Disorders, European Medicines Agency, Amsterdam, Netherlands; 2 Unit Neurology, Psychiatry, Ophthalmology, Federal Institute of Drugs and Medical Devices, Bonn, Germany; 3 Department of Experimental and Clinical Pharmacology, Medical University of Warsaw; 4 The Office for Registration of Medicinal Products, Medical Devices and Biocidal Products, Warsaw, Poland; 5 Medicines Evaluation Board, Pharmacotherapeutic group 1, Utrecht, Netherlands; 6 French National Agency for Medicines and Health Products Safety (ANSM), Saint-Denis, France; 7 European Medicines Agency’s Central Nervous System Working Party (CNSWP)

**Keywords:** Depression, medicines, Clinical trial, innovation, EMA

## Abstract

According to the World Health Organization (WHO), depressive disorders are currently considered as one of the most disabling medical conditions in the world with one of the highest disability-adjusted life years [1] and this situation has apparently been further worsened during the COVID-19 pandemic [2]. Up to two thirds of patients with major depressive disorders (MDD) do not achieve full remission following an adequate first line standard of care and/or experience residual symptoms such as anxiety, impaired cognition, fatigue, sleep disturbance, or anhedonia [3]. Several attempts are often needed to find the most suitable treatment [4]. Thus, there is a need for medicinal products with better efficacy (e.g., faster onset of action, higher rates of response and remission), improved safety and/or more personalised profiles [5].

According to the World Health Organization (WHO), depressive disorders are currently considered as one of the most disabling medical conditions in the world with one of the highest disability-adjusted life years [1] and this situation has apparently been further worsened during the COVID-19 pandemic [2]. Up to two thirds of patients with major depressive disorders (MDD) do not achieve full remission following an adequate first line standard of care and/or experience residual symptoms such as anxiety, impaired cognition, fatigue, sleep disturbance, or anhedonia [3]. Several attempts are often needed to find the most suitable treatment [4]. Thus, there is a need for medicinal products with better efficacy (e.g., faster onset of action, higher rates of response and remission), improved safety and/or more personalised profiles [5].

The European Medicines Agency (EMA) has recently released a revised draft Guideline on clinical investigation of medicinal products in the treatment of depression for external consultation till 31 March 2024 [6]. This general guidance document replaces the previous EMA guideline (EMA/CHMP/185423/2010 Rev. 2, which came into force in 2013) on the development of medicinal products for acute and long-term treatment of major depressive episodes that occur in the context of MDD. Revision 3 of the guideline also addresses possible extrapolations of data on the treatment of depression associated with bipolar and related disorders.

The guideline is divided into several sections, each covering a different topic related to the development of products for the treatment of depression. It starts with an introduction of the disease including information on the epidemiology, pathophysiology, and classification of depressive disorders. Then, it describes the general principles and objectives of clinical trials of antidepressant drugs and provides methodological considerations on the study design, including notably the selection of study population, the choice and measurement of outcome variables, and the most common targets of estimation (i.e., estimand) that need to be aligned with trial planning, design, conduct, analysis, and interpretation. The typical design to demonstrate efficacy and safety of an antidepressant is a randomized, double-blind, placebo-controlled, parallel-group study comparing change in the primary endpoint. The use of an active comparator would still be helpful to put the results into context, but it is no longer considered to be mandatory for licensing and therefore not discussed in the revised guideline. However, the results, besides being statistically significant, must be comprehensive and clinically meaningful. This requires the incorporation of rates of response/remission to adequately assess clinical relevance. Generally, two trials are needed to allow adequate evaluation of short-term efficacy. Furthermore, it must also be shown that the initial response to treatment of the index episode is maintained in at least one study. Attention is drawn to several challenges in the clinical development for antidepressants and notably how to tackle the high placebo effect. Use of a placebo run-in period and subsequent patient selection are considered problematic with regards to the generalisability of the results.

The guideline also discusses the most relevant scientific question(s) of interest and corresponding estimands for clinical trials of antidepressant drugs. These must be clearly pre-specified, including the intercurrent events and their handling strategy. Specific considerations regarding the statistical methods and analysis plan aligned to the estimand are outlined. Handling of missing data is of particular concern, as substantial amount of missing data is to be expected based on trial results from the past.

This revision 3 of the guideline also reconsiders the requirements for clinical trials in difficult to treat patient populations (treatment resistant and partial responders). The previous distinction between add-on or augmentation trials in partial responders versus monotherapy trials for non-responders is no longer considered valid, since efficacy for treatment-resistant depression (TRD) has been shown in an add-on setting with the approval of esketamine (Spravato [7]). In the previous version, TRD was considered as failure of at least two different first-line antidepressants prescribed in adequate dosages for optimal duration and with satisfactory affirmation of treatment adherence. However, according to the revised guideline, the inclusion of patients with one failed treatment at a maximum tolerated dose and of adequate duration is now considered an option to represent the population of patients with TRD. Furthermore, retrospective assessment of partial response or lack of response is also considered an acceptable approach to select this population, provided it is based not only on the patient’s recollection of symptom improvement, but also on medical records.

As regards the emergence and requirements for antidepressants with a rapid onset of effect, double-blind, randomised, parallel group, placebo-controlled clinical trials are also needed, as for conventional antidepressants. Depending on the mechanism and assumed onset of action, an earlier efficacy endpoint could be appropriate, if measured accordingly with a validated assessment tool. Maintenance of effect will also have to be characterised in at least one study with an appropriate design.

In terms of recent innovation in the field, the revised guideline highlights the research and development for the repurposing of psychedelics [8, 9]. It is acknowledged that the clinical development of psychedelic agents presents several challenges, such as use of placebo, and risk of unblinding, choice of comparator, expectancy bias, dosing, safety, maintenance of effect, and role of associated psychotherapy or psychological support [10, 11]. However, as with all other antidepressants, randomized, double-blind, placebo-controlled, short-term trials are needed to establish a positive benefit/risk, as well as trials to confirm the maintenance of effect. It is recommended to start clinical investigation in a more severely affected population, such as patients with TRD [12].

Taking into consideration the advances in the field, several other important updates have been made. Development of targeted therapies to address symptom clusters, which persist despite current treatment, such as claims to improve cognition, sleep, or anhedonia are also discussed. However, the patient population studied should not be artificially narrowed and a pathophysiological justification for the claimed mechanisms of action to treat specific symptoms is required. Dedicated trials designed to test the specific hypothesis of efficacy in the context of a separate symptom, domain, or dimension are required using validated and pertinent tools as well as adequate endpoints. The effect must be demonstrated in addition to – and independently from – the improvement of depressive symptoms. Furthermore, the efficacy in the targeted (cluster of) symptoms should be specific for depression and not applicable to the same (clustered) symptoms in other conditions. Similarly, the revised guideline also includes a section with several specifiers for depressive disorders, such as the diagnostic features “anxious distress” and “with peripartum onset,” as per the Diagnostic and Statistical Manual of Mental Disorders (DSM-5). If a claim for such a sub-population is pursued, a dedicated trial with specific inclusion criteria and appropriate endpoints is required.

Other issues related to gender and special populations, including the elderly and paediatric population are also addressed in the revised guideline. Possible gender and drug metabolism differences [13, 14] in MDD [15] are emphasised with the recommendation to perform analyses of gender-specific groups to allow an estimate of potential differences. Furthermore, the need for studies on efficacy and safety of antidepressants in children and adolescents is highlighted, with possible extrapolation from adult data for maintenance of effect and long-term efficacy, provided that robust and comparable evidence of short-term efficacy has been shown in both adults and the paediatric population.

In terms of safety, updates have been made to draw attention to potential concerns in special populations and antidepressants with new mechanisms of action. The importance of monitoring suicidal thoughts and behaviour under antidepressant therapy by use of validated instruments is emphasized.

Overall, the guideline has been revised to reflect the latest advances and innovations in the field of research and development for MDD, including the emergence of new therapies and the need to foster more personalised treatments. The aim of the revised guideline is to harmonise the standards and requirements for clinical trials of antidepressant drugs in the European Union and thereby to facilitate the development and approval of new treatment options. The ongoing external consultation will help to discuss the outstanding clinical and methodological challenges to promote development and innovation in the field of MDD.

The European Medicines Agency’s (EMA) Central Nervous System Working Party (CNSWP) role is to carry out specific tasks such as preparing, reviewing and updating guidelines and concept papers related to central nervous system issues. Central Nervous System Working Party | European Medicines Agency (europa.eu).
